# *In vivo* development of elevated linezolid resistance mediated by a deletion in ribosomal protein L3 in clinical *Enterococcus faecium*

**DOI:** 10.1128/aac.00871-25

**Published:** 2025-10-28

**Authors:** Lingyan Sun, Tailong Lei, Yeqiong Liu, Shanshan Lu, Xiaoting Hua, Junxiong Zhang, Yan Chen

**Affiliations:** 1 Department of Laboratory Medicine, The First Affiliated Hospital, Zhejiang University School of Medicine 667770, Hangzhou, China; 2 Zhejiang Key Laboratory of Clinical In Vitro Diagnostic Techniques, Hangzhou, China; 3 Institute of Laboratory Medicine, Zhejiang University 12377, Hangzhou, China; 4 Department of Infectious Diseases, Sir Run Run Shaw Hospital, Zhejiang University School of Medicine 56660, Hangzhou, China; 5 Key Laboratory of Microbial Technology and Bioinformatics of Zhejiang Province, Hangzhou, China; 6 Regional Medical Center for National Institute of Respiratory Diseases, Sir Run Run Shaw Hospital, Zhejiang University School of Medicine 56660, Hangzhou, China; 7 Hangzhou Linping Traditional Chinese Medicine Hospital, Hangzhou, China; Shionogi Inc., Florham Park, New Jersey, USA

**Keywords:** linezolid resistance, ribosomal protein L3, *Enterococcus faecium*, *rplC*

## Abstract

Gram-positive bacteria can develop resistance to linezolid through mutations in the 50S large-subunit ribosomal proteins, such as L3. However, this mechanism is rarely reported in clinical enterococcal strains. Here, we describe a pair of *Enterococcus faecium* strains, 1505efm and 1583efm, isolated from a meningioma patient at different stages of linezolid treatment. Strain 1583efm, which was obtained after 17 days of linezolid treatment, exhibited an eightfold higher linezolid MIC (64 mg/L) than the initially isolated strain 1505efm (8 mg/L). Meanwhile, 1583efm became susceptible to erythromycin, tetracycline, and high-level streptomycin due to the loss of plasmid-borne resistance genes *erm(A)*, *erm(B)*, *tet(M)*, *ant(6)-Ia*, and *aph(3′)-III*. Whole-genome comparison revealed a deletion in the *rplC* gene of 1583efm, resulting in the loss of 21 amino acids (∆Tyr139-Arg159) in ribosomal protein L3. Structural modeling showed that this deletion disrupts the linezolid hydrogen bond network near the ribosomal peptidyl transferase center, reducing its binding affinity. The mutation, which remained stable over 50 generations without antibiotics, imposed a significant fitness cost on the bacterial cells and led to an increase in the expression of the truncated *rplC* gene. Our study uncovers a novel linezolid resistance mechanism involving the deletion of ribosomal protein L3 in clinical *E. faecium* strains within the host.

## INTRODUCTION

Linezolid (LZD) is an important clinical option for treating serious gram-positive infections, especially multidrug-resistant strains such as methicillin-resistant *Staphylococcus aureus*, vancomycin-resistant enterococci, and multidrug-resistant *Streptococcus pneumoniae* (1, 2). It exerts its antibacterial effect by binding to the ribosomal peptidyl transferase center (PTC) of the 50S subunit, thereby inhibiting bacterial protein synthesis. However, shortly after its introduction into clinical use in 2000, LZD-resistant strains emerged from clinical *Enterococcus faecium* and *Staphylococcus aureus* (3, 4) and subsequently were reported in coagulase-negative staphylococci (5) and other species (6).

Ribosomal mutations in domain V of 23S rRNA, especially G2576U, have been reported as the primary mechanisms of LZD resistance among clinical isolates of enterococci and staphylococci and are particularly prevalent in Europe and the United States (7, 8). Besides, mobile oxazolidinone resistance genes, such as *cfr* and *cfr*-like genes encoding 23S rRNA methyltransferases, as well as *optrA* and *poxtA*, which code for members of the ABC-F protein family, have been identified as being responsible for resistance to oxazolidinones and phenicols (9–14). In China, acquisition of *optrA* is the predominant resistance mechanism observed in clinical enterococci, particularly in *Enterococcus faecalis*, and typically mediates low-level resistance to LZD (15, 16).

In addition, mutations in 50S large-subunit ribosomal proteins, particularly ribosomal protein L3 (RPL3) and ribosomal protein L4, which are located close to the PTC, have also been found to be associated with LZD resistance (17). Mutations in RPL3 are often associated with resistance to both oxazolidinones and pleuromutilins (e.g., tiamulin and retapamulin), as their binding sites in the PTC overlap (18, 19). Previous reports on RPL3 mutations related to oxazolidinones resistance have primarily been documented in staphylococci (20–23), but such mutations in enterococci have rarely been reported. This study revealed a novel mechanism of LZD resistance caused by a deletion in RPL3 (∆Tyr139-Arg159) by comparing a pair of in vivo-evolved *E. faecium* strains. The study also investigated the phenotypic stability and fitness cost associated with this deletion. Furthermore, we explored the underlying mechanism by which the ∆Tyr139-Arg159 mutation contributes to LZD resistance through analysis of the LZD-bound 50S crystal structure.

## MATERIALS AND METHODS

### Strains and clinical data

**Fig 1:**
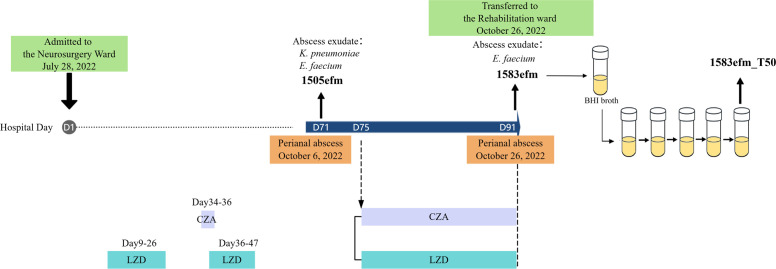
Strain isolation timeline and related patient’s antimicrobial treatment course. Strain 1583efm_T50 was obtained through 50 generations of continuous *in vitro* culture without antibiotics to test the stability of the resistance phenotype of strain 1583efm. During the patient’s hospitalization, pathogens such as *Klebsiella pneumoniae*, *Serratia marcescens*, *Enterobacter cloacae*, and *Pseudomonas aeruginosa* were isolated from purulent discharge of the surgical incision on the head, urine, and sputum. From 28 July to 6 October, the patient was treated with two courses of LZD (a total of 30 days) and 3 days of ceftazidime-avibactam (CZA), in addition to other antimicrobial agents including caspofungin, vancomycin, levofloxacin, piperacillin-sulbactam, and meropenem. To highlight the main focus of this study, this figure only lists the pathogens isolated from the perianal abscess site and the relevant antibiotic treatment cycles.

A pair of sequential clinical *E. faecium* isolates were obtained from a 68-year-old inpatient admitted to the neurosurgical ward of a tertiary hospital of Hangzhou due to recurrence after meningioma resection. The timeline of strain isolation, which is shown in Fig. 1, provides detailed information. The first strain of *E. faecium*, designated as 1505efm, was isolated from the perianal abscess purulent discharge on 6 October 2022. Strain 1505efm exhibited low-level resistance to LZD with a MIC of 8 mg/L. After the patient underwent 17 days of treatment with LZD and ceftazidime-avibactam from 10 to 26 October 2022, the second strain of *E. faecium*, named 1583efm, was isolated from the perianal purulent exudate on 26 October 2022. Strain 1583efm was high-level resistant to LZD (64 mg/L). Species identification of the two strains was initially carried out via the Bruker Biotyper MALDI-TOF MS system (Bruker Daltonics, Bremen, Germany), with subsequent validation through whole-genome sequencing.

### Antimicrobial susceptibility testing

The MICs of 11 drugs were performed using the agar dilution method. Results were interpreted according to the standards from Clinical and Laboratory Standards Institute M100, 35th Edition. Results for tigecycline were interpreted according to the European Committee on Antimicrobial Susceptibility Testing Clinical Breakpoint Tables v.15.0 for enterococci. *E. faecalis* American Type Culture Collection (ATCC) 29212 and *S. aureus* ATCC 25923 were used as the quality control strains.

### *Sma*I-pulsed-field gel electrophoresis

Agarose plugs of different isolates were prepared for pulsed-field gel electrophoresis (PFGE) as previously detailed (24). *Sma*I enzyme-digested restriction DNA fragments were separated using the CHEF-DR II system (Bio-Rad, USA) under the conditions of clamped homogeneous electric field for 22 h at 6 V/cm, 120° switch angle, 3–20 s switch time, and 14°C in 0.5% Tris-borate-EDTA electrophoresis buffer. *Xba*I-digested DNA of *Salmonella enterica* serotype Braenderup strain H9812 was used as a molecular size marker.

### Whole-genome sequencing and analysis

Strains 1505efm and 1583efm were given whole-genome sequencing on the Illumina NovaSeq 6000 (Illumina Inc., San Diego, CA, USA) and the MinION platform (Nanopore, Oxford, UK).

The genomes of strains 1505efm and 1508efm were assembled using Nanopore long reads with RAVEN v.1.1.10 (25), followed by polishing with Illumina short reads using Polypolish v.0.5.0 (26). The assembled contigs were annotated via the RAST web server (27) and subsequently refined manually with the National Center for Biotechnology Information BLASTP programs. Resistance genes, MLST types, and plasmid replicons were identified with default settings using the ResFinder 4.6.0, MLST 2.0, and PlasmidFinder 2.1 web databases, respectively (http://www.genomicepidemiology.org). Sequence alignment and visualization were carried out with Proksee web server v.1.1.4 (28).

### Stability testing of the resistance

We cultured strain 1583efm successively on antibiotic-free medium to achieve the isolate after 50 generations, referred to as 1583efm_T50 (Fig. 1). The specific procedures are as follows: after overnight growth of single colonies in 2 mL of brain-heart infusion (BHI) broth, the cultures were continued for five additional overnight periods (2 µL inoculated into 2 mL of BHI broth, 10 generations per day) without antibiotics (Fig. 1). The MICs of LZD for strain 1583efm_T50 were determined to assess the stability of LZD resistance of 1583efm.

### Transcriptional analysis by reverse transcription quantitative PCR (qRT-PCR)

Reverse transcription PCR was employed to assess the relative gene expression levels of *optrA* and *rplC*. Total RNA extraction from cultures in the same mid-log phase (5.5 h for 1505efm and 3.5 h for 1583efm) was performed with an RNeasy Mini Kit (Qiagen GmbH, Germany). Reverse transcription of RNA samples was conducted to prepare cDNA samples using a PrimeScript RT reagent kit (Takara Bio, Japan).

Real-time quantitative PCR was carried out in a 20 µL reaction as described previously (29). The 2^−ΔΔCt^ method was used to analyze the data from three independent experiments. The housekeeping gene *purK* served as an internal control. Designed primers for *optrA*, *rplC*, and *purK* are presented in Table S1.

### Growth measurement

Bacterial growth rates were assessed using a Bioscreen C MBR (Oy Growth Curves AB Ltd., Helsinki, Finland) as described previously (30). The R package “growthrates” was used to determine the coefficient of determination (*r*²), the lag-phase duration (lag.t), and the specific growth rate (*m*) during the exponential phase. Based on the formula *m* = ln 2 / Td, the doubling time (Td) was then calculated.

### Structure modeling and visualization

Following sequence alignment of the *rplC* gene from strain 1505efm, the cryo-EM structure of *E. faecalis* 70S ribosome (Protein Data Bank entry: 7NHK) was selected as the structure template for modeling the *rplC*-encoded RPL3 in this study. However, since the absence of LZD in this structure, the LZD bound *E. coli* 70S ribosome structure (Protein Data Bank entry: 7S1H) was employed to contextualize the LZD binding environment through structural alignment of RPL3. The P-site fMet-tRNA from 7NHK was retained in the model. The RPL3 model from strain 1583efm was mutated from the strain 1505efm template, followed by stepwise energy minimization. Subsequent to protein modeling, all complexes underwent global preprocessing and refinement using the OPLS3e force field (31). Structure visualization and analysis were implemented by PyMOL 3.1.

## RESULTS

### Antimicrobial susceptibility and genome comparison of 1505efm and 1583efm

To analyze the homology between strains 1505efm and 1583efm, we first performed PFGE using genomic DNA digested with the enzyme *Sma*I. As shown in Fig. S1, the two strains shared an identical PFGE type, indicating their high degree of homology. Furthermore, single-nucleotide polymorphism (SNP) analysis was conducted between these two strains using Snippy 4.4.5 to reveal their evolutionary relationships. It revealed that the genomes of the two strains differ by 6 variant-SNPs, 1 variant-deletion, and 4 complex variants. These findings suggest that strain 1583efm evolved from strain 1505efm, indicating a high degree of homology between them.

When comparing the antimicrobial susceptibility phenotypes of the two strains (Table 1), we found that strain 1583efm exhibited a significantly higher level of resistance to LZD (MIC = 64 mg/L) compared with strain 1505efm (MIC = 8 mg/L), representing an eightfold increase in MIC. Besides, strain 1505efm was resistant to tetracycline (MIC = 32 mg/L), erythromycin (>64 mg/L), and high concentrations of streptomycin, whereas strain 1583efm became susceptible to tetracycline (MIC = 0.25 mg/L), erythromycin (<0.125 mg/L), and high concentrations of streptomycin.

**TABLE 1:** Antimicrobial susceptibility of strains 1505efm and 1583efm^*a*^^,^^*b*^

Strains	MIC (mg/L)												
	LZD	CL	AMP	VA	CIP	LEV	FOS	RIF	TET	E	TGC	High-STR	High-GEN
1505efm	**8**	32	>128	1	>64	64	128	1	**32**	**>64**	0.125	**R**	S
1583efm	**64**	32	>128	1	>64	64	128	1	**0.25**	**<0.125**	0.06	**S**	S

^*a*^ AMP, ampicillin; CIP, ciprofloxacin; CL, chloromycetin; E, erythromycin; FOS, fosfomycin; high-GEN, high-concentration gentamicin; high-STR, high-concentration streptomycin; LEV, levofloxacin; LZD, linezolid; R, resistance; RIF, rifampin; S, susceptible; TET, tetracycline; TGC, tigecycline; VA, vancomycin.

^*b*^ The antimicrobial MIC values that have changed significantly are marked in bold. Tigecycline MIC dropped from 0.125 mg/L to 0.06 mg/L, a non-significant change.

**Fig 2:**
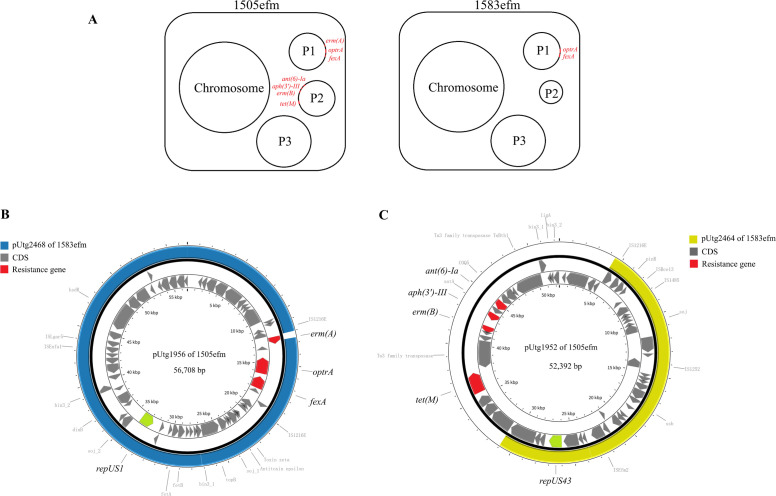
Genomic comparison between 1505efm and 1583efm (**A**) and pairwise alignment for the plasmids with the same replicon type generated by Proksee (**B and C**). (**A**) Schematic diagram of the overall genomic composition. (**B**) Comparison of plasmid_1 (P1): plasmid pUtg1956 (accession number CP189598) in 1505efm and pUtg2468 (accession number CP189713) in 1583efm. (**C**) Comparison of plasmid_2 (P2): pUtg1952 (accession number CP189601) in 1505efm and pUtg2464 (accession number CP189714) in 1583efm.

To understand the reason, we conducted an in-depth analysis of the whole-genome data of the two strains. Strains 1505efm and 1583efm both belong to the ST1693 type. Both strains possess a chromosome of approximately 2,774 kb in size and contain three circular plasmids (Fig. 2; Table S2). By contrast, there is a notable difference in the size of the plasmids with the replicon repUS43. As shown in Table S2, the plasmid pUtg2464 carried by strain 1583efm is only 26,539 bp in size, which is about half the size of the pUtg1952 plasmid in strain 1505efm (52,392 bp). As regards the resistance genes, we found that both of their chromosomes harbor *dfrG*, *msr(C)*, and *aac(6′)-Ii*. However, compared with strain 1505efm, strain 1583efm lost five plasmid-borne resistance genes: *erm(A)*, *ant(6)-Ia*, *aph(3′)-III*, *erm(B*), and *tet(M*). It can be reasonably inferred that the absence of five genes, including *erm(A*) and *erm(B*) that mediate macrolide resistance, *tet(M*) that mediates tetracycline resistance, and *aph(3′)-III* and *ant(6)-Ia* that mediate aminoglycoside resistance, are responsible for the 1583efm strain becoming susceptible to erythromycin, tetracycline, and high-level streptomycin.

We carried out a comparative analysis of the plasmids of the same replicon type between the two strains. By comparing the plasmids with the replicon repUS1, pUtg2468 of strain 1583efm shares 100% similarity and 99% coverage with pUtg1956 of strain 1505efm (Fig. 2A). Both of them carry *fexA* and *optrA*, which mediate oxazolidinone resistance. The protein encoded by *optrA* is a new OptrA variant that differed in five amino acid exchanges (T112K, S147L, Y176D, A324E, and T481P) compared to the wild type (accession number KP399637). The only discrepancy between the two plasmids is that pUtg2468 of strain 1583efm has lost a macrolide resistance *erm(A*).

About replicon repUS43, pUtg2464 in the genome of 1583efm shared 100% similarity and 51% coverage with pUtg1952 in the genome of 1505efm (Fig. 2B). That means plasmid pUtg2464 has lost nearly half of its nucleotide sequence, and it just happens that four resistance genes [*ant(6)-Ia*, *aph(3′)-III*, *erm(B)*, and *tet(M)*] are located in that region.

**Fig 3:**
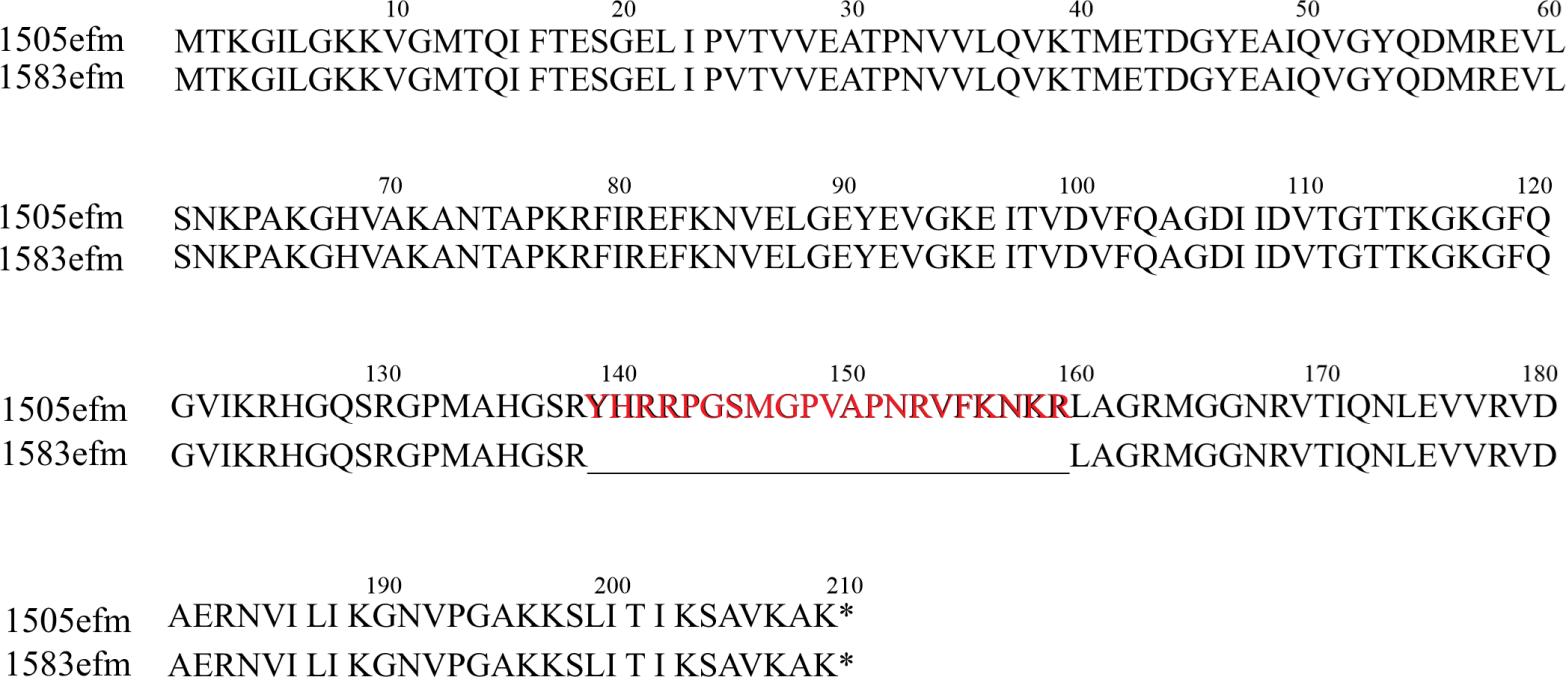
Alignment of the amino acid sequences of 50S ribosomal protein L3 between 1505efm and 1583efm. The letters in the figure represent the abbreviations of amino acids, and the numbers above the letters indicate the positions of the amino acids. The red-marked positions from 139 to 159 represent the 21-amino acid sequence that is missing in 1583efm. The “*” at the end indicates the stop codon.

Then, we performed a comparative analysis between the chromosomes of two strains based on the detailed analysis of the variants provided by Snippy 4.4.5. Only a meaningful variant, a 63 bp deletion (variant DEL) on the chromosome of strain 1583efm was identified to be seated on the gene *rplC* encoding 50S ribosomal protein L3. The *rplC* at position from 928803 to 928174 on the chromosome in 1505efm is 630 bp in length, while *rplC* at positions 827,985–828,551 in 1583efm is only 567 bp. A 63-bp deletion (nucleotides 415–477) in the *rplC* gene, encoding the 50S ribosomal protein L3, results in the loss of 21 amino acids: YHRRPGSMGPVAPNRVFKNKR (Fig. 3). Mutations in *rplC* have been reported previously as a significant mechanism for the decreased susceptibility of gram-positive strains to LZD (22). Thus, it is reasonable to speculate that the 63-base (corresponding to 21 amino acids) deletion in *rplC* identified in this study is the key factor for the increased LZD resistance of strain 1583efm. Subsequently, we conducted structure modeling and comparative analyses of ribosomal protein L3 in both strains to elucidate the reason for the significant increase in LZD resistance in the 1583efm strain.

### Structural insights into *rplC*-mediated alterations in LZD-ribosome interactions

**Fig 4:**
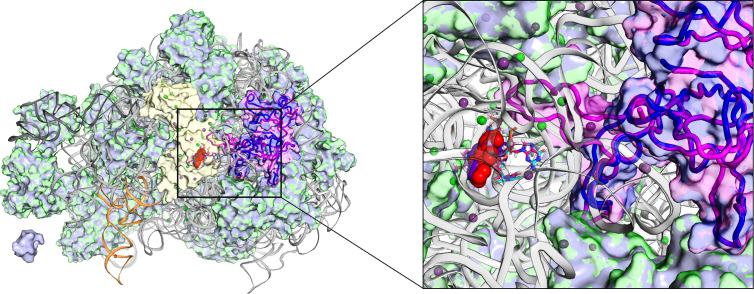
Binding mode comparison of LZD bound 50S ribosomal subunit. Superposition of the structure of LZD bound 50S ribosome from strains 1505efm and 1583efm. RPL3 is magenta; RPL3^mut^ is blue; rRNAs are gray; P-site fMet-tRNA is orange; LZD is red; other proteins are slate gray overlaid on green, magnesium ions and potassium ions are depicted as small green and purple spheres.

To elucidate the impact of the *rplC* mutation on LZD resistance in *E. faecium*, we constructed models of the LZD-bound ribosome before and after the mutation. In 2008, the archaeal and bacterial crystal structures (3CPW and 3DLL) confirmed the localization of the LZD binding pocket within the PTC of the 50S ribosomal subunit, a finding previously suggested by mutational and cross-linking studies. As illustrated in Fig. 4, LZD occupies the aminoacyl position of tRNA in the A-site and thereby blocks protein synthesis. It forms multiple hydrogen bonds extensively with many 23S rRNA nucleotides in the PTC and is stabilized by magnesium coordination. Wild-type RPL3 in strain 1505efm extends its N- and C-terminal domains over the surface of the 50S subunit, with a central loop projecting into the PTC, whereas the mutant RPL3 in strain 1583efm deletes this loop sequence (Fig. 4). As shown in Fig. S2, the loop deletion disrupts local interactions within the PTC, potentially altering the conformation of residues (particularly G2505 and U2506) and weakening hydrogen bonds with LZD. This rearrangement may also hinder transpeptidation by weakening LZD binding to key residues in peptidyl transfer, including A2451 (32, 33) and U2504 (33). Owing to the current limitations of modeling algorithms, definitive predictions of precise structural changes are not feasible; nevertheless, our modeling allows a tentative prediction that deletion of RPL3 would likely induce rearrangement of the LZD-binding pocket, thereby reducing its affinity for the ribosome.

### Stability and fitness cost of the deletion

To test the stability of the deletion in *rplC* in strain 1583efm, we serially passaged 1583efm for 50 generations in antibiotic-free medium to obtain strain 1583efm_T50.

There was no change in LZD MIC between 1583efm and 1583efm_T50 as determined by the E-test, suggesting no phenotypic reversion. Additionally, by amplifying the *rplC* gene of 1583efm_T50 via PCR and conducting Sanger sequencing, we found it to be consistent with that of strain 1583efm. This indicates that the deletion in 1583efm is stable at both the genetic and phenotypic levels.

To observe whether this deletion brought a fitness cost to the strains, we conducted growth assays on the three strains. Compared with strain 1505efm (0.76 h), the doubling times of strains 1583efm and 1583efm_T50 were significantly increased (1.11 h). Besides, the lag phases were also prolonged from 2.7 to 4.5 h. This indicates that the *rplC* mutation imposes a substantial fitness cost on the mutant strain.

### Gene copy number and transcriptional level for *optrA* and *rplC*

To determine whether the copy number and expression level of *optrA* play a role in the high-level resistance to LZD in strain 1583efm, we compared the copy number and relative mRNA level of *optrA* between strains 1505efm and 1583efm. The copy number of *optrA* was estimated as the ratio of the median read coverage of *optrA* to that of *purK*. As Table S3 shows, the estimated copy numbers of *optrA* in the genomes of strains 1505efm and 1583efm were 2.28 and 1.67, respectively, showing no significant difference.

**Fig 5:**
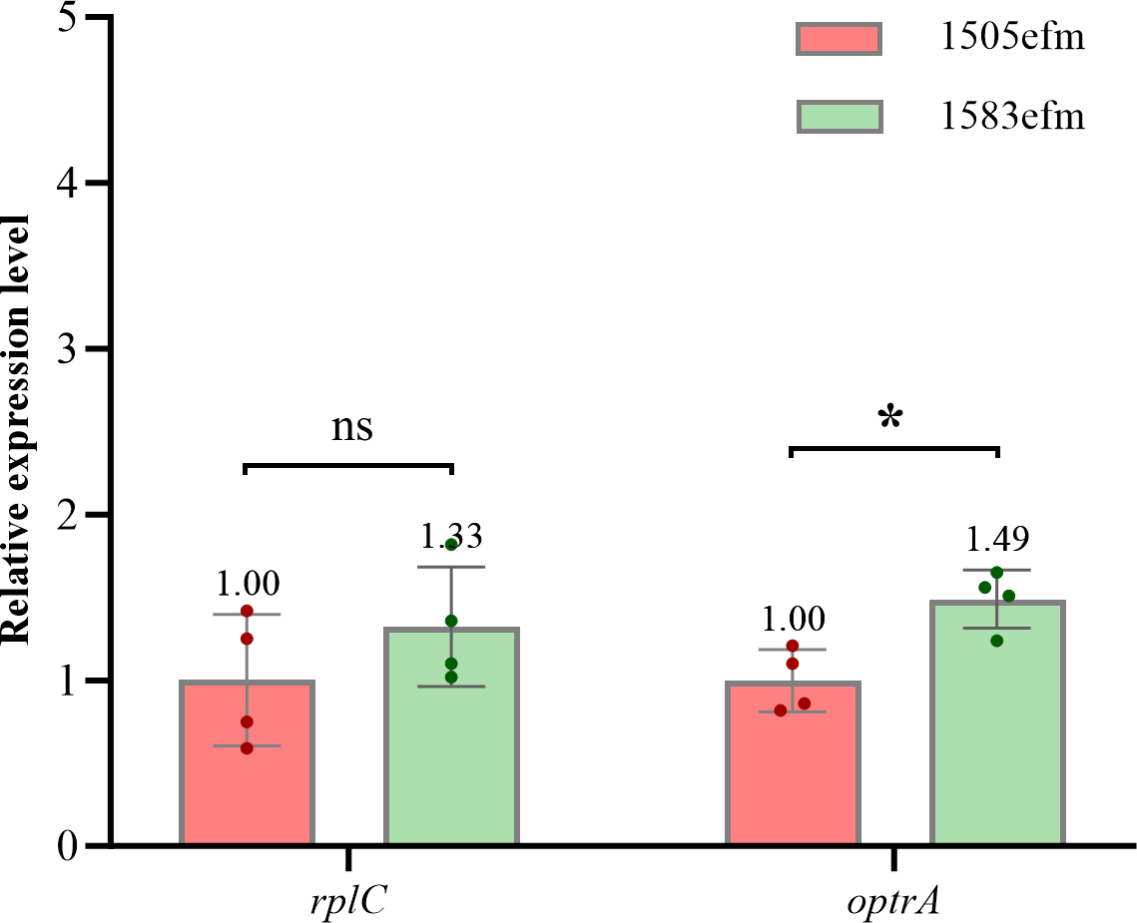
Relative expression level of the gene *optrA* and *rplC* for strains 1505efm and 1583efm using quantitative PCR. 1505efm was used as the reference strain, and *purK* was used as the reference gene. All experiments were conducted with four biological replicates, each consisting of four technical replicates. Error bars represent the standard error of the mean. Statistical analysis was performed through unpaired Mann-Whitney *U* test. *, *P* < 0.05. ns, not significant.

However, the expression level of *optrA* in strain 1583efm was slightly higher (1.49-fold) than that in strain 1505efm (*P* < 0.05) (Fig. 5). In addition, to determine whether the deletion in *rplC* affects its transcription, we compared the mRNA levels of *rplC* in the two strains, and there was no significant difference in *rplC* expression between strains 1505efm and 1583efm (Fig. 5).

## DISCUSSION

In recent years, there has been an increasing trend in the isolation of LZD non-susceptible clinical isolates of enterococci. As indicated by data from global antimicrobial resistance surveillance programs such as ZAAPS and SENTRY, alterations in 23S rRNA remain the primary oxazolidinone resistance mechanism in *E. faecium*, while the *optrA* gene is more prevalent in *E. faecalis* (34, 35). In China, clinical enterococcal isolates are predominantly *E. faecalis*, which typically carry the *optrA* gene that usually mediates low-level resistance (15, 16). Notably, carriage of the *optrA* gene is also the main resistance mechanism among the clinical *E. faecium* isolates in China (36). This is mainly because *optrA* is typically located on a plasmid, which makes it more likely to be transferred horizontally, thereby facilitating its widespread dissemination in clinical enterococci. In this study, both strains 1505efm and 1583efm contained plasmid-borne *optrA* genes. Earlier case-control studies have identified prior exposure to LZD—especially prolonged courses—as a key risk factor for linezolid resistance (37, 38). In this case, prior to this hospitalization, the patient had been hospitalized for 13 days (29 June–12 July 2022) for a craniotomy and meningioma resection, during which LZD was administered prophylactically from 1 to 11 July 2022. In addition, the patient had received two further courses of linezolid, totaling approximately 30 days before the 1505efm strain was isolated in the present hospitalization. This cumulative LZD exposure likely contributed to the development of low-level resistance in the 1505efm strain via acquisition of the *optrA* gene.

Interestingly, strain 1505efm developed high-level resistance to LZD during LZD treatment *in vivo*, yet it became susceptible to erythromycin, tetracycline, and high-level streptomycin due to the loss of plasmid-borne resistance genes *erm(A)*, *erm(B)*, *tet(M)*, *ant(6)-Ia*, and *aph(3′)-III*. The loss of resistance genes on the plasmid is not uncommon, and it can occur both within patients and during the laboratory strain passaging process. However, from another perspective, this may be a manifestation of collateral sensitivity (39). Abandoning resistance to other antimicrobial agents in order to adapt to LZD pressure might be an active evolutionary strategy employed by bacteria in this study.

Previous reports of mutations in ribosomal 23S rRNA or proteins in clinical isolates were usually associated with prolonged LZD treatment (40, 41). Similarly, the RPL3 mutation in strain 1583efm emerged after the patient had undergone 17 days of LZD therapy in this study. Not all RPL3 mutations are associated with reduced LZD susceptibility; rather, mutations occurring in the region close to the PTC have been shown to endow with a resistance effect. Reported point mutations in enterococci (e.g., S113L, C369T, T600C, and C606T) co-occur with *optrA* and have not been validated in an *optrA*-negative background, leaving their individual impact unresolved (15, 16). The G4K/D/R, T150A mutations of RPL3 have been detected in several *optrA*-positive but LZD-susceptible clinical enterococcal strains and are therefore not considered relevant for LZD resistance (42). In staphylococci, previously reported RPL3 mutations associated with resistance are almost all located within the region near the PTC of positions 137–169, such as G137A/D, G139R, H146R/Q, F147L, G152D, G155R, A157R, S158Y, and M169L (43–47). Upon comparing the RPL3 amino acid sequences between *S. aureus* and *E. faecium*, we discovered that the 139th to 159th amino acids of RPL3 in *E. faecium* correspond to the positions from 147 to 168 of RPL3 in *S. aureus*. From the structure of the RPL3 protein, the deleted region from 139 to 159 is precisely the area of RPL3 that is closest to the PTC (Fig. 3) in our study. Compared to point mutations, deletions of several amino acids are more likely to cause significant rearrangements in the secondary and tertiary structures of RPL3. The ∆Tyr139-Arg159 deletion in RPL3 distorts the PTC cavity, akin to the collapse of a suspended spring roof. This deformation disrupts the hydrogen bond network of LZD and reduces its binding affinity.

In the mutant strain, we also observed a slight increase in the expression level of the *optrA* gene (Fig. 5). The deletion in RPL3 may not confer high-level LZD resistance on its own but rather potentiate the resistance conferred by the ribosome protection protein OptrA. Notably, as depicted in Fig. S3, the distance between OptrA and the LZD-binding pocket in the RPL3-deletion ribosome is greater than 20 Å, which exceeds the typical range for direct allosteric interaction. This spatial separation suggests that the RPL3 deletion is unlikely to directly enhance OptrA’s activity through conformational changes. Instead, the deletion may create a more permissive microenvironment within the ribosome, such as altering the local electrostatic potential or RNA structure, thereby potentiating OptrA’s intrinsic capacity to remove LZD from the RPL3-deletion ribosome.

Beyond ribosome structure, LZD binding also involves interactions with the nascent peptide chain, particularly the penultimate alanine residue, which influences drug affinity and context-dependent specificity (48, 49). As illustrated in Fig. S3, the observed configuration closely resembles the actual crystal structure (48), with the nascent chain positioning its alanine residue toward the second benzene ring of LZD, forming an unconventional CH-π interaction exceeding the typical distance threshold (≤3.5 Å) (50, 51). Our analysis further reveals steric clashes between alanine’s methyl group and the first morpholine ring of LZD. These structural anomalies likely arise from the constraints of the superimposed model, which primarily depicts relative positioning rather than absolute conformation. Nonetheless, our data indicate that stalling residues such as alanine or other methyl-containing residues positioned opposite LZD’s benzene ring can effectively impair ribosomal translocation. Although our current focus centers on ribosomal structural changes, these ancillary molecular interactions probably collectively contribute to the observed LZD resistance phenotype.

**Fig 6:**
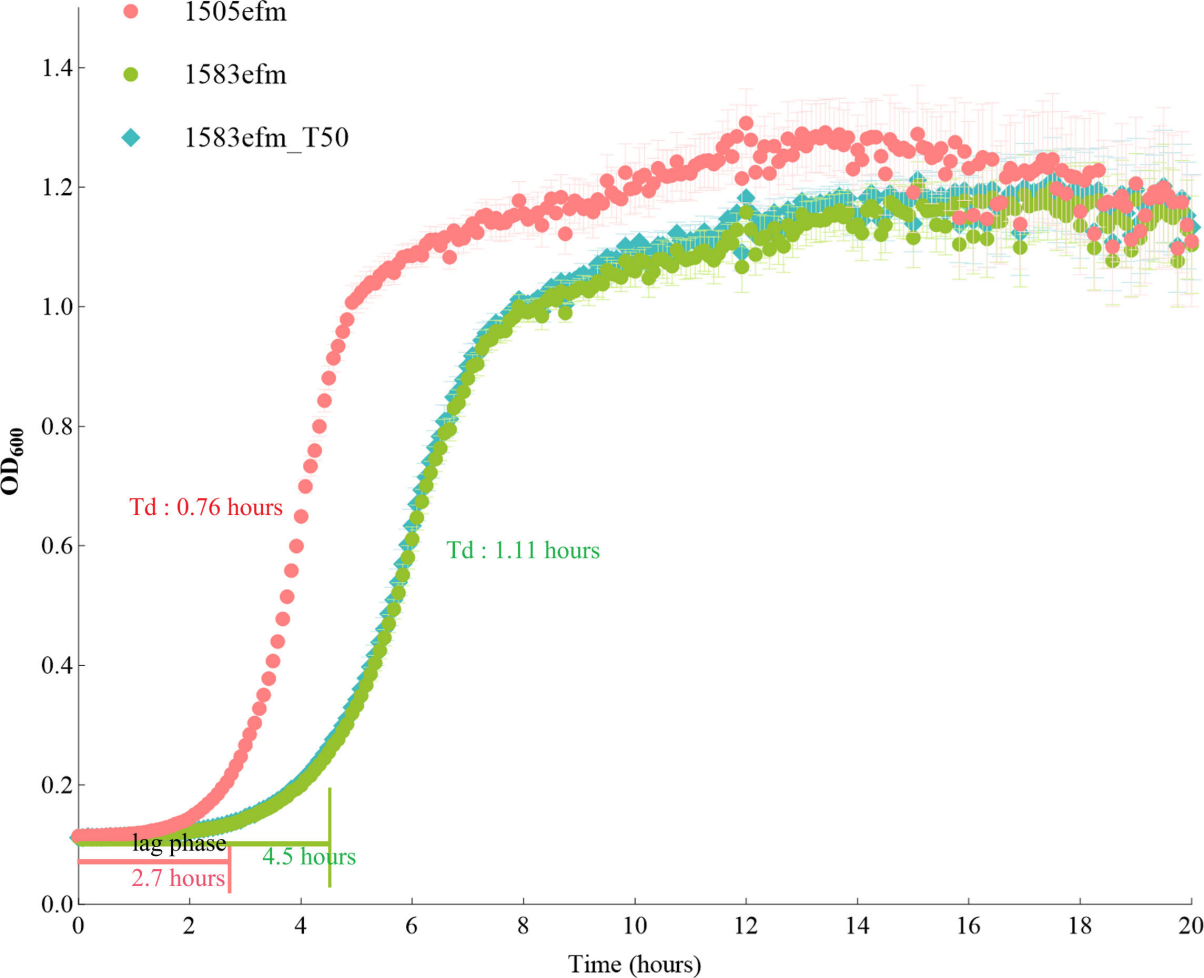
Growth curves for strains 1505efm, 1583efm, and 1583efm_T50 cultured in medium without antibiotics. The R package “growthrates” was used to determine the coefficient of determination (*r*²), the lag-phase duration (lag.t), and the specific growth rate (*m*) during the exponential phase. The doubling time (Td) was calculated based on the formula *m* = ln 2/Td.

Besides, a significant fitness cost in the mutant 1583efm was observed in our study (Fig. 6). Previous research has demonstrated that the majority of mutations in RPL3 tend to cause only slight increases in bacterial doubling time. However, a small number of mutations, such as G144D and G147R in RPL3, have been shown to result in significant growth impairments (22). Our structural modeling has shown that the ∆Tyr139-Arg159 deletion in the mutant RPL3 induces local collapse of the PTC. According to the literature, deletions or mutations at key residues of RPL3 are generally associated with decreased peptidyl-transferase activity (52). Once the defective RPL3 is incorporated into the ribosome, the catalytic efficiency of the PTC declines; the rate of peptide-bond formation slows; and the overall translational elongation rate drops markedly, manifesting as a reduced growth rate (53). This conjecture may partly explain the fitness cost associated with the mutation.

In short, this study reports an increase in LZD resistance due to a deletion of Tyr139-Arg159 in the ribosomal protein L3 in clinical isolates of enterococci under *in vivo* LZD pressure. This novel RPL3 mutation has not been previously described in enterococci. This study offers novel insights into the mechanisms of resistance to LZD and provides theoretical support for the rational use of antibiotics in clinical practice.

## Data Availability

Complete sequences of the chromosome and plasmids from 1505efm and 1583efm have been deposited in GenBank under accession numbers CP189598–CP189601 and CP189712–CP189715.

## SUPPLEMENTAL MATERIAL

Supplemental material aac.00871-25-s0001.docx Tables S1 to S3; Fig. S1 to S3.

ASM does not own the copyrights to Supplemental Material that may be linked to, or accessed through, an article. The authors have granted ASM a non-exclusive, world-wide license to publish the Supplemental Material files. Please contact the corresponding author directly for reuse.
