# Supplementary material for: In vivo development of elevated linezolid resistance mediated by a deletion in ribosomal protein L3 in clinical Enterococcus faecium
Source: Antimicrob Agents Chemother. 2025 Oct 28;69(12):e00871-25. doi: 10.1128/aac.00871-25 (PMC12691684; doi:10.1128/aac.00871-25)
Supplement: Supplemental material — Tables S1 to S3; Fig. S1 to S3. [file aac.00871-25-s0001.docx]

Table S1 Primers designed in this study

| Primer | DNA sequence (5´ to 3´) | Tm (℃) | Length of target (bp) |
| --- | --- | --- | --- |
| Q-rplC-F | GAATTCACGAATGAAGCGCTTA | 55 | 157 |
| Q-rplC-R | TCCAAACGTAGTACTACAAGTG | 53 |  |
|  |  |  |  |
| Q-optrA-F | CGAATAGTGTATCATCTCCACTGA | 55 | 155 |
| Q-optrA-R | TGTTGGGAGTAATGGTATTGGTA | 54 |  |
|  |  |  |  |
| Q-purK-F | GATATCCAAGATGCGATTGACG | 55 | 154 |
| Q-purK-R | CTTCTAAAACACAGGTTCCTTCTC | 54 |  |
|  |  |  |  |
| rplC-intact-F | GAATAATGCTACATTCGGCATTC | 54 | 753 |
| rplC-intact-R | AAGCTAGACTTACCATCTGGTG | 54 |  |

Table S2. The genomic characteristics of 1505efm and 1583efm

| Strains | ST | Genome | Plasmid replicon | Sizes (bp) | Resistance gene |
| --- | --- | --- | --- | --- | --- |
| 1505efm | 1693 | Chromosome | NA | 2,774,767 | *dfrG, msr(C), aac(6')-Ii* |
|  |  | Plasmid_1: pUtg1956 | repUS1 | 56,708 | *fexA, optrA, erm(A),* |
|  |  | Plasmid_2: pUtg1952 | repUS43 | 52,392 | *ant(6)-Ia,* *aph(3')-III, erm(B), tet(M)* |
|  |  | Plasmid_3: pUtg1948 | repUS15 | 149,547 | ND |
| 1583efm | 1693 | chromosome | NA | 2,774,839 | *dfrG, msr(C), aac(6')-Ii* |
|  |  | Plasmid_1: pUtg2468 | repUS1 | 56,147 | *fexA, optrA,* |
|  |  | Plasmid_2: pUtg2464 | repUS43 | 26,539 | ND |
|  |  | Plasmid_3: pUtg2466 | repUS15 | 148,219 | ND |

NA: Not Applicable；ND: Not Detected.

Table S3. Estimation of *optrA* copy number by mapping NGS reads

| Strains | Genes | The number of mapped reads^a^ | The average length of mapped reads^b^ (bp) | The length of target sequence^c^ (bp) | Mean sequence coverage^d^ | Estimated gene copy no. |
| --- | --- | --- | --- | --- | --- | --- |
| 1505efm | *purK* | 1829.00 | 145.17 | 1134.00 | 234.14 | 1.00 |
|  | *optrA* | 7152.00 | 146.98 | 1968.00 | 534.15 | **2.28** |
| 1583efm | *purK* | 2026.00 | 144.68 | 1134.00 | 258.48 | 1.00 |
|  | *optrA* | 5828.00 | 145.48 | 1968.00 | 430.82 | **1.67** |

For a specific gene, d = ab/c; As a housekeeping gene on the chromosome, the copy number of *purK* is assumed to be 1 by default. Estimated gene copy no. of *optrA =* d*^optrA^/* d*^purK^.*


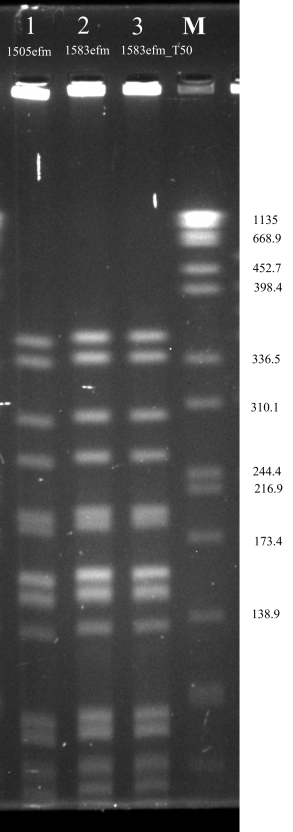


**Figure S1 DNA patterns in different isolates by *Sma*I-PFGE.** *Xba*I-digested DNA of *Salmonella enterica* serotype Braenderup strain H9812 was used as a molecular size marker. The sizes of the molecular marker (M) are indicated. Lanes 1–3: 1505efm; 1583efm; 1583efm_T50.


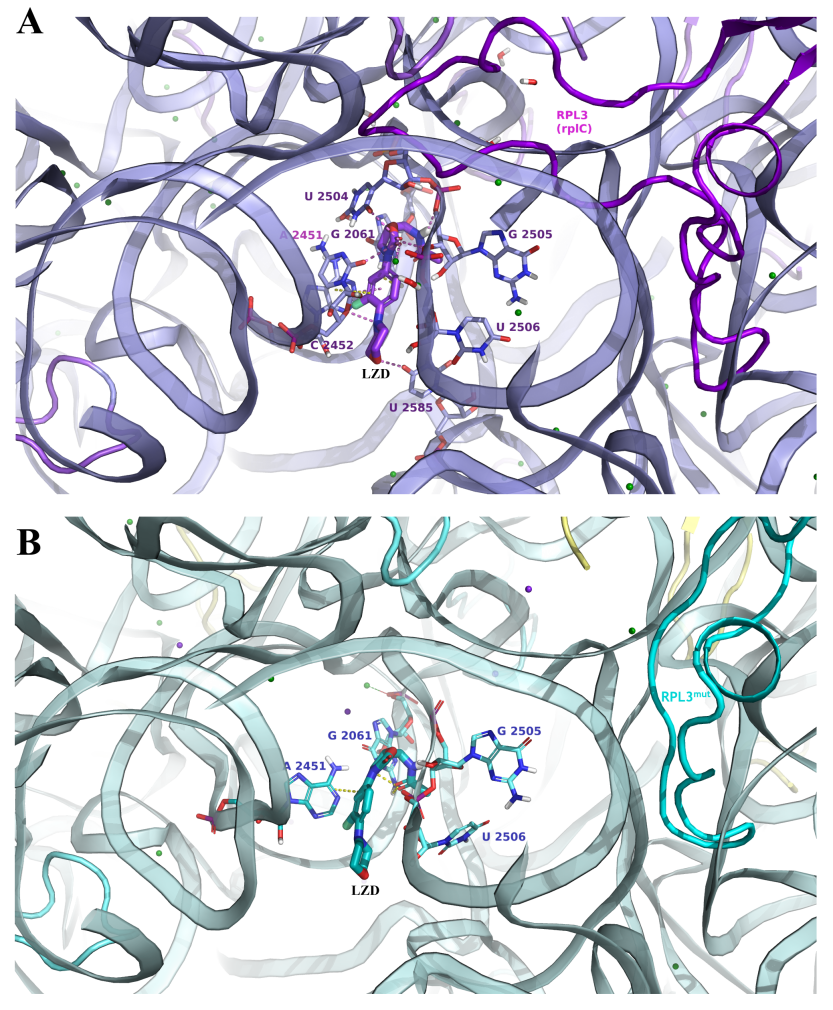


**Figure S2.** **Local comparison of LZD-ribosome interactions between the strains 1505efm and 1583efm.** (A) Local view of LZD-ribosome interactions and adjacent RPL3 from the strain 1505efm, where all non-bonded interactions are shown in violet dash lines. (B) Local view of LZD-ribosome interactions and adjacent RPL3^mut^ from the strain 1583efm, where all non-bonded interactions are shown in yellow dash lines. The RNA and protein chains are numbering with uppercase letters.

**
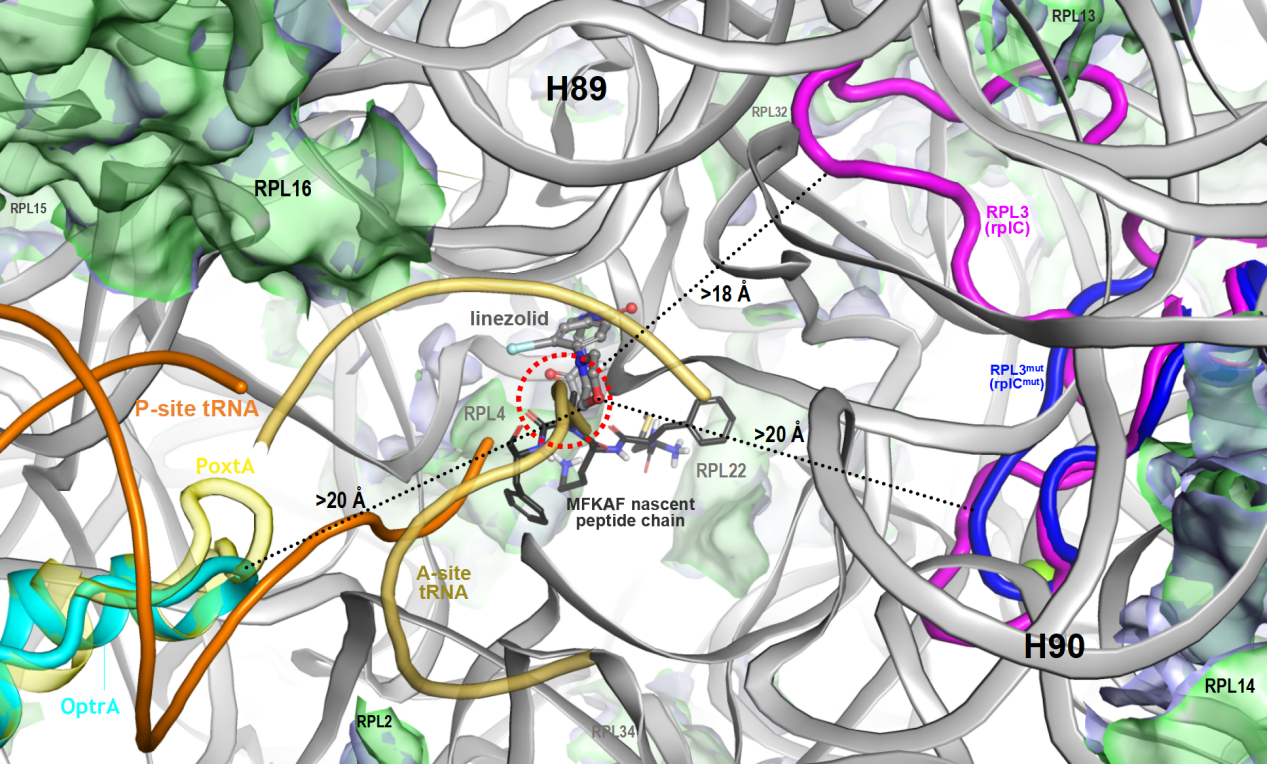
**

**Figure S3. The penultimate alanine of the nascent peptide chain clashes with LZD, while OptrA has no direct interaction with LZD.** It shows the relative spatial positions of superimposed OptrA, PoxtA, ribosomal proteins, tRNAs, and LZD-bound ribosomes. The 23S rRNA from the strain 1583efm is shown as gray ribbon, OptrA is shown as cyan ribbon, PoxtA is shown as paleyellow ribbon, ribosomal proteins from the strain 1505efm is shown as purple surface, ribosomal proteins from the strain 1583efm is shown as green surface, RPL3 from the strain 1505efm is shown as magenta loop, RPL3 from the strain 15835efm is shown as blue loop, P-site tRNA is shown as orange loop, A-site tRNA is shown as olive loop, LZD is shown as stick with gray carbon, the nascent peptide chain is shown as stick with black carbon.The main RNA loops and ribosomal proteins are labeled with the numbering names.

## **Structure modeling of OptrA**

Following sequence alignment of *optrA* gene from the strain 1505efm, the cryo-EM structure of *E. faecalis* 70S ribosome (PDB ID: 7P7T) was selected as the structure template. The overall structure was first aligned with our modeled LZD-bound ribosome. Successively, the protein PoxtA within the 7P7T structure was extracted and used as a template for modeling the resistance protein OptrA in this study. Subsequent to protein modeling, all complexes underwent global preprocessing and refinement using the OPLS3e force field (1). Finally, both the OptrA model and the PoxtA structure were superimposed onto the modeled LZD-bound ribosome to map relative spatial position. Additionally, the nascent peptide chain and P-site tRNA from PDB 7S1G, along with the A-site tRNA from PDB 7P7T, were superimposed onto the LZD-bound ribosome model for comparative analysis (RMSD is 0.488 Å and 0.663 Å respectively). All structure visualization and analysis were implemented by PyMOL (2).
